# Short-term effects of a park-based group mobility program on increasing outdoor walking in older adults with difficulty walking outdoors: the Getting Older Adults Outdoors (GO-OUT) randomized controlled trial

**DOI:** 10.1186/s12877-024-05331-4

**Published:** 2024-09-06

**Authors:** Nancy M. Salbach, Nancy E. Mayo, Sandra C. Webber, C. Allyson Jones, Lisa M. Lix, Jacquie Ripat, Theresa Grant, Cornelia van Ineveld, Philip D. Chilibeck, Razvan G. Romanescu, Susan Scott, Ruth Barclay

**Affiliations:** 1 Department of Physical Therapy, Rehabilitation Sciences Institute, University of Toronto, 160-500 University Avenue, Toronto, ON M5G 1V7 Canada; 2 The KITE Research Institute, Toronto Rehabilitation Institute - University Health Network, Toronto, ON Canada; 3 School of Physical and Occupational Therapy, McGill University, Montreal, QC Canada; 4 Department of Physical Therapy, University of Manitoba, Winnipeg, MB Canada; 5 Department of Physical Therapy, University of Alberta, Edmonton, AB Canada; 6 Department of Community Health Sciences, Max Rady College of Medicine, University of Manitoba, Winnipeg, MB Canada; 7 Department of Occupational Therapy, University of Manitoba, Winnipeg, MB Canada; 8 Bruyère Research Institute, Ottawa, ON Canada; 9 Section of Geriatric Medicine, University of Manitoba, Winnipeg, MB Canada; 10 College of Kinesiology, University of Saskatchewan, Saskatoon, SK Canada; 11 McGill University Health Centre Research Institute, Montreal, QC Canada

**Keywords:** Older adults, Outdoor walking, Physical activity, Randomized controlled trial, Task-oriented training, Community exercise program, Parks, Walking capacity, Walking self-efficacy

## Abstract

**Background:**

We estimated the short-term effects of an educational workshop and 10-week outdoor walk group (OWG) compared to the workshop and 10 weekly reminders (WR) on increasing outdoor walking (primary outcome) and walking capacity, health-promoting behavior, and successful aging defined by engagement in meaningful activities and well-being (secondary outcomes) in older adults with difficulty walking outdoors.

**Methods:**

In a 4-site, parallel-group randomized controlled trial, two cohorts of community-living older adults (≥ 65 years) reporting difficulty walking outdoors participated. Following a 1-day workshop, participants were stratified and randomized to a 10-week OWG in parks or 10 telephone WR reinforcing workshop content. Masked evaluations occurred at 0, 3, and 5.5 months. We modeled minutes walked outdoors (derived from accelerometry and global positioning system data) using zero-inflated negative binomial regression with log link function, imputing for missing observations. We modeled non-imputed composite measures of walking capacity, health-promoting behavior, and successful aging using generalized linear models with general estimating equations based on a normal distribution and an unstructured correlation matrix. Analyses were adjusted for site, participation on own or with a partner, and cohort.

**Results:**

We randomized 190 people to the OWG (*n* = 98) and WR interventions (*n* = 92). At 0, 3, and 5.5 months, median outdoor walking minutes was 22.56, 13.04, and 0 in the OWG, and 24.00, 26.07, and 0 in the WR group, respectively. There was no difference between groups in change from baseline in minutes walked outdoors based on incidence rate ratio (IRR) and 95% confidence interval (CI) at 3 months (IRR = 0.74, 95% CI 0.47, 1.14) and 5.5 months (IRR = 0.77, 95% CI 0.44, 1.34). Greater 0 to 3-month change in walking capacity was observed in the OWG compared to the WR group (βz-scored difference = 0.14, 95% CI 0.02, 0.26) driven by significant improvement in walking self-efficacy; other comparisons were not significant.

**Conclusions:**

A group, park-based OWG was not superior to WR in increasing outdoor walking activity, health-promoting behavior or successful aging in older adults with difficulty walking outdoors; however, the OWG was superior to telephone WR in improving walking capacity through an increase in walking self-efficacy. Community implementation of the OWG is discussed.

**Trial registration:**

ClinicalTrials.gov NCT03292510 Date of registration: September 25, 2017.

**Supplementary Information:**

The online version contains supplementary material available at 10.1186/s12877-024-05331-4.

## Background

Promoting active living [1] in older adults is a global priority. People aged 65 and over are a burgeoning portion of the population that is expected to rise from 10% in 2022 to 16% in 2050 worldwide [2]. The number of women surpasses the number of men in each age category over the age of 65 years due to an advantage in life expectancy [2]. Healthcare and municipal authorities must build an infrastructure of community programs and services promoting active living to prevent mobility decline and the onset of frailty that are driven by comorbidity as people age. For example, almost 75% of older adults aged 65 years and older with arthritis report a limitation in walking a mile with a higher prevalence of severe limitation in women compared to men [3]. In contrast, the prevalence of mobility limitation in the general population of older adults is approximately 40% [4].

Approximately a third of older adults walk outdoors fewer than 3 days a week [5], a phenomenon exacerbated by the COVID-19 pandemic [6]. Limited outdoor walking is associated with not only a decline in mobility, functional independence, social engagement, and health-related quality of life (HRQL) [7, 8] but also increased healthcare utilization [9]. Limited outdoor walking is an indicator of frailty [10] and frailty is associated with a high risk of falls, hospitalization, and death in older adults [11]. Approximately 44.2% of older adults are pre-frail [12] which indicates an opportunity for intervention. Frailty models identify limitations in physical capacity, such as walking speed, and physical activity [13], as frailty indicators; thus, targeting these outcomes may slow the onset of frailty.

There is growing interest in developing and implementing community-based programs to increase exercise participation and physical activity among people with chronic disabling health conditions [14, 15] and older adults in general [16, 17]. Community-based programs involving outdoor physical activity in natural environments may be superior to indoor programs in promoting moderate-to-vigorous physical activity [18] and enhancing mental health [19]. Group programs involving practice of outdoor mobility tasks, such as walking uphill, over uneven terrain (e.g., grass, gravel), and at different speeds, may confer superior health benefits compared with individual walking practice indoors due to the greater physical, cognitive, and social demands [20, 21]. Despite the need to practice moving in outdoor environments, few interventions involving the practice of mobility tasks in an outdoor environment have been developed to promote successful aging in older adults [22]. Outdoor interventions evaluated to date have included unsupervised use of outdoor walking trails with graded intensity levels [23]; supervised group walking on a circular track, incorporating balance exercise while walking, combined with indoor resistance training [24]; supervised and progressive group Nordic pole walking, including uphill and downhill walking, in parks [25]; and supervised and progressive group walking incorporating band gymnastics, dual-task-based exercise, and walking on varied terrain in an urban forest [26]. Improvement in gait speed, lower limb function, and strength were observed following walking on a circular track combined with resistance training [24], and group Nordic pole walking in parks improved lower limb strength but not balance and mobility compared to indoor circuit training [25]. Despite the importance of outdoor walking activity, studies have not targeted outdoor walking as an outcome. Use of theoretical frameworks to inform development of these programs, which aim to foster behaviour change, is recommended [27]. Theory can be used to conceptualize study outcomes, identify strategies for behaviour change, and highlight individual and environmental factors that may influence intervention effects (i.e., effect modifiers). Thus, theory can directly inform intervention design, selection of variables, and interpretation of findings. Evaluations of outdoor walking interventions, however, have not reported use of theories of behaviour change or conceptual frameworks of community ambulation.

We developed and piloted a theory-based group, supervised, program incorporating park-based task-oriented training of community mobility to increase outdoor walking activity and promote successful aging [28]. We used a model of community mobility [29] suggesting that outdoor walking can be achieved by building skills and self-efficacy in eight dimensions: distances, temporal factors, ambient conditions, physical load, terrain, attentional demands, postural transitions, and traffic density to guide program design. This model [29] also posits that outdoor walking results from an interaction between individual factors, such as walking capacity, income, and car access, and environmental factors, such as neighbourhood walkability [30]. After establishing the safety, feasibility, and acceptability of our trial protocol [28], we planned a rigorous evaluation [31] that we report on here to help justify implementation in the community.

Our primary objective was to estimate the short- and long-term effects of a 1-day educational workshop followed by a 10-week, task-oriented outdoor walk group (OWG) program compared to the workshop and subsequent 10 telephone weekly reminders (WR) program, on increasing outdoor walking activity in older adults with difficulty walking outdoors. We hypothesized that the change in number of minutes per week spent walking outdoors from baseline to 3, 5.5, and 12 months, would be significantly higher in individuals who complete the OWG program than in those who complete the WR program.

Secondary objectives were to: (1) estimate the extent to which sex, initial frailty level, and intervention dose received, modified the effect of the OWG compared to the WR on increasing outdoor walking activity; and (2) estimate the short- and long-term effects of the OWG compared to the WR on improving secondary outcomes including walking capacity; health-promoting behavior; and successful aging.

## Methods

### Study design and oversight

The Getting Older Adults Outdoors (GO-OUT) study (registered 25/09/2017 with ClinicalTrials.gov NCT03292510) was an evaluator-masked, four-site, two-parallel-group randomized controlled trial with a one-to-one allocation ratio. Details of the full trial protocol have been previously reported [31]. The research ethics boards at participating sites approved the study protocol. An independent data safety monitoring board met quarterly to review adverse event and falls data. We used strategies, such as setting numerical limits for data entry, and reviewing site-specific summaries of data and comments entered, to optimize quality of data entry into REDCap data management software [32]. Study biostatisticians and lead researchers verified the accuracy and completeness of the data and statistical analyses. We used CONSORT guidance [33, 34] to inform reporting.

### Eligibility criteria and recruitment

The target population was older adults with difficulty walking outdoors. Criteria for study inclusion were: age 65 years or older, difficulty walking outdoors, living independently in the community, ability to walk one block (~ 50 m) independently with or without a walking aid, limited time (< 75 min/week) spent walking outdoors during good weather months from May to October (note: this criterion was dropped for the second cohort as participants had difficulty responding), willingness to sign a waiver or obtain physician clearance to exercise, mental competency indicated by a minimum score of 18 on the Mini-Mental State Exam telephone version [35], availability to participate in the workshop and at least 5 weeks of the OWG program, and ability to speak and understand English. Criteria for exclusion were: meeting physical activity guidelines of 150 min per week, receiving rehabilitation to improve walking, or at high risk for falls defined as meeting at least one of the following criteria: [36] a) ≥ 2 falls in the last year or presents with an acute fall; b) health conditions preventing safe and full participation; c) postural hypotension (defined as a drop in systolic blood pressure of > 20 mmHg or a drop in diastolic blood pressure of > 10 mmHg taken after lying supine for 5 min to after standing for 2 min; d) resting heart rate < 45 or > 100 beats per minute; and e) severe limitation in visual acuity (i.e., self-reported difficulty, while wearing usual eyewear, with reading the newspaper or distinguishing a person’s facial features from across a room [37]). We gave participants the option to participate with a family member/friend who met the study eligibility criteria so that our interventions better reflected real-world community programs. We used community-based strategies, such as advertising with newspapers, radio stations, senior’s centres, residences, and organizations, and health condition-specific organizations, to recruit individuals in Edmonton, Winnipeg, Toronto, and Montreal, Canada. Site coordinators screened for eligibility and enrolled participants.

### Interventions and randomization

The theoretical foundation and details of the interventions have been previously reported [31]. Two cohorts of individuals, recruited in 2018, and 2019, respectively, were invited to participate over a 12-month period. The intervention period was timed to occur during summer months (i.e., June-August) across sites because interventions involved promotion and/or practise of walking outdoors. Following baseline evaluations, each site scheduled participants for a workshop.

#### Workshop

Participants were asked to attend a one-day, interactive educational workshop prior to randomization. Workshops occurred at community centres or university locations in their respective city. At the beginning of the workshop, participants received a pedometer for personal use and a booklet that included information and worksheets reviewed during the workshop. Participants circulated in groups of 2–3 to eight activity stations that covered (1) Canadian physical activity guidelines; (2) setting specific, measurable, achievable, realistic, and timed (SMART) goals; (3) use of pedometers; (4) Nordic pole walking; (5) walking patterns, foot care, and footwear; (6) prevention of falls; (7) exercising safely, including how to monitor exercise intensity; and (8) balance exercises and posture. Activities at each station were designed to increase knowledge, self-efficacy, and skill in areas that would promote safe outdoor walking behaviour. A student in a health-related program or a health professional with experience working with older adults, with cardiopulmonary resuscitation certification, served as a facilitator at each station. Facilitators were trained and provided with standardized protocols for running each station. After the workshop, site coordinators used REDCap software [32] to stratify participants by site and participant type (enrolment as an individual or with a partner) and randomize them in a concealed manner in randomly ordered blocks of 2 or 4 to the experimental intervention (i.e., OWG) or the active control intervention (i.e., WR group). A biostatistician external to the research team prepared the random allocation sequence.

#### Outdoor walk group

The OWG program was designed to build skill and self-efficacy to walk outdoors through progressive, task-specific practice. Across sites, walk groups were run at large parks, accessible by public and adapted transport, with features (e.g., walking pathways, hills) required for the walking program, frequent rest locations, washroom and parking facilities, and scenic aspects (e.g., gardens).

The OWG program consisted of two, 1-hour sessions per week for 10 weeks. A maximum of nine participants and three facilitators per group was allowed to achieve a 3:1 participant-to-facilitator ratio to optimize safety and permit subgrouping based on ability level. Each session included a 10-minute warmup, a continuous distance walk, task-oriented practice of an outdoor walking skill, a second continuous distance walk, and a 10-minute cool down. Activities during the two sessions in the same week were the same. Activities were designed to build competence in dimensions of mobility [29], including distance, postural transitions, temporal factors, physical load, attentional demands, traffic density (e.g., walking in a crowded area), terrain, and ambient conditions. For example, week 1 involved practice of distance walking to address the distance dimension, and walking and turning, stepping sideways, starting and stopping and standing up from a bench on an outdoor walking path, to address the postural transitions dimension. Walking activities had two levels of difficulty for people with a comfortable gait speed of < 0.8 m/s vs. ≥ 0.8 m/s to allow tailoring to ability level. Walking task difficulty gradually increased over the 10-week period.

The OWG leader was a health professional (e.g., physical therapist, kinesiologist) with experience working with people with chronic health conditions. The leader directed each session with help from one to two assistants with training in a health-related program. Leaders completed a 2-hour training session and were provided with participant contact and health information. Leaders and assistants received a program guide (outlining roles and responsibilities of lead and assistant facilitators, session cancellation criteria, equipment, program principles, safety guidelines for assistants, and detailed protocols for weekly activities). Specific guidelines provided leaders with instructions on when to cancel sessions due to high heat, wind, precipitation, or poor air quality.

#### Weekly reminders

In the WR program, site coordinators telephoned participants weekly for 10 weeks, with the option to deliver the content by email or during the phone call the following week if the person was unavailable. Reminders were scripted and required participants to review and discuss information in the workshop workbook, including outdoor walk goals and strategies to prevent falls [38].

### Outcomes and measures

Trained evaluators, masked to intervention assignment, conducted in-person evaluations in university and community settings using standardized protocols at baseline, 3 months (immediately post-intervention), 5.5 months, and 12 months. The primary outcome was change from baseline to each follow-up time point in the number of minutes spent walking outdoors derived from accelerometry and global positioning system (GPS) data. At each evaluation, evaluators provided participants with an accelerometer (ActiGraph GT3X + activity monitor (ActiGraph, Pensacola, FL)), and a GPS monitor (Qstarz BT-Q1000XT A-GPS Travel Recorder) affixed to a belt. Evaluators instructed participants to position the devices on the belt over the right hip during waking hours for eight consecutive days. Subsequently, study personnel retrieved devices from people’s homes or, in some cases, participants returned the devices by mail. At each evaluation timepoint, we included data for individuals who wore the devices a minimum of 10 h per day for at least four days [39]. GT3X + monitors were initialized to collect data in 1s epochs. GT3X + nonwear time included intervals of ≥ 90 consecutive minutes with zero activity counts (allowing for up to two consecutive minutes with counts between 0 and 100) [40]. Walking bouts ≥ 5 min in duration with cadence levels ≥ 40 steps/min were identified in the ActiGraph data (low frequency extension (LFE) files) [41]. Walking bout location was then manually identified in Google Earth Pro by latitude/longitude coordinates associated with walking bouts. If the walking bouts occurred on city streets (non-residential area) and/or in neighborhoods (residential areas) or parks (outdoor paths, green spaces, golf courses), they were designated as outdoor walking. As part of the planned process evaluation [31], we asked participants to wear accelerometers and GPS monitors during a single OWG session in week 3 and week 9 to measure walking speeds and distances achieved during the two continuous distance walks.

Secondary outcomes were change from baseline to each follow-up time point in walking capacity, health-promoting behavior, and successful aging. Each secondary outcome was derived from scores on multiple measures as these measures captured overlapping constructs. Improvement on each secondary outcome was defined as statistically significant improvement on any one of the underlying measures. Walking capacity was derived from five measures that were selected based on an expectation of task-specific training effects. These five measures included the 14-item Mini Balance Evaluation Systems test [42, 43] (mini-BESTest) scored from 0 to 28 points (higher scores reflect better balance); 30-second sit-to-stand test [44, 45] (30STS) scored as the number of sit-to-stands completed in 30 s; 10-metre walk test [46] (10mWT) that measures comfortable walking speed in metres per second (m/s); 6-minute walk test [47] (6MWT) that documents distance in metres walked on a straight, 30-metre walkway in six minutes; and the ambulatory self-confidence questionnaire [48] (ASCQ) scored from 0 to 10 points (higher scores reflect high self-confidence). Health-promoting behavior was derived from two measures, including moderate-to-vigorous physical activity minutes/day derived from accelerometry data (cut point ≥ 760 counts per minute) [49, 50]; and the life space assessment (LSA) questionnaire [51] scored from 0 (totally bed-bound) to 120 (travels out of the city every day without assistance) [52]. Successful aging is a multidimensional concept of which engagement in meaningful activities, and well-being are primary components [53–55]. Thus, successful aging was derived from three measures, specifically the community health activities model program for seniors [56] (CHAMPS) questionnaire, scored as hours spent in meaningful activity per week; and the RAND-36 [57] emotional well-being scale and RAND general health item, each scored from 0 to 100 where higher scores represent the most optimal health state. At each follow-up evaluation, we also documented participation in co-interventions (e.g., physical therapy) with potential to influence walking activity.

### Participant characteristics

At each timepoint, we administered the Cardiovascular Health Study Frailty Index [11] to identify the presence of five frailty indicators: (1) unintentional weight loss in the last year; (2) exhaustion; (3) low physical activity; (4) slow walking speed, and (5) weak grip strength. Level of frailty was then classified based on the number of indicators present: frail (3–5 indicators present); pre-frail (1–2 indicators present); not frail (no indicators present) [11].

At baseline, neighbourhood walkability was assessed using the self-report neighbourhood environment walkability scale [58, 59] (NEWS) that produces 8 multi-factor subscales and 5 single factor subscales [59, 60]. Subscale scores can range from 173 to 865 for the residential density subscale, 1 to 5 for the land use mix diversity subscale, and 1 to 4 for the remaining 11 subscales (access to services, streets in neighbourhood, places for walking and cycling, neighbourhood surroundings, traffic hazards, neighbourhood safety, lack of parking, lack of cul-de-sacs, hilliness, physical barriers, and social interaction). At baseline, we measured height and collected self-report data on comorbidity using the Charlson Comorbidity Index [61, 62] (CCI), weight, age, sex, education, marital status, employment, income, smoking status, use of glasses, use of mobility devices, car access, medications, and reasons for outdoor walking limitation.

### Adverse events

We described our procedures for monitoring occurrence of falls and adverse events in the study protocol [31]. We defined a serious adverse event as a fall or injury leading to persistent or significant disability or incapacity that lasted more than 48 h and resulted in limited activities of daily living, hospitalization, or death [63, 64]. Non-serious adverse events included falls leading to no injury or minor injury, cuts, and shortness of breath. A data safety monitoring board consisting of three academic researchers not associated with the study, with expertise in falls and rehabilitation, met quarterly to review data on falls and adverse events. Our stopping guideline [31] was based on falls occurrence. Our guideline indicated that if the rate of falls that occurred during study evaluations, study interventions or while walking outdoors for exercise, and resulted in a serious adverse event exceeded 7% of the total number of participants randomized in the study, then the data safety monitoring board and research team would discuss stopping the trial.

### Sample size

Details of our sample size calculation for the primary analysis have been reported [31]. Based on our pilot study results [28], we expected an effect size (ES) of 0.5 for the 0 to 3-month and 0 to 5.5-month comparisons (based on a between-group difference in outdoor walking time of 25 min per week, SD = 50), and a smaller ES, due to the effect of weather, of 0.4 for the 0 to 12-month comparison (based on a between-group difference in outdoor walking time of 20 min per week, SD = 50). Sample size estimation was based on detecting the smaller ES of 0.4. We also accounted for a 5% attrition rate from 0 to 6 months, and a 20% attrition rate from 0 to 12 months, based on rates observed in studies of group-based physical activity interventions [65]. Given an ES of 0.4, a Type I error level of 0.05, a Type II error level of 0.20, equal number of participants/group, and a 20% attrition rate, a total sample size of 240 (120 per group) was required.

### Statistical analysis

Due to precautions related to the COVID-19 pandemic [66], we were unable to collect data on the primary outcome measure and secondary performance-based outcome measures from participants in cohort 2 at 12 months post-baseline to evaluate long-term intervention effects as originally planned [31]. Thus, we limited our analysis to an evaluation of short-term intervention effects based on data collected at 0, 3, and 5.5 months.

We modeled the primary study endpoint using a zero-inflated negative binomial regression with log link function [67]. First, the sum of minutes of outdoor walking across all wear days was created and entered as the dependent variable in the model. The log of the number of valid wear days during which the accelerometry and GPS devices were worn was included as the offset in the model. This is equivalent to modeling the number of minutes walked outside per day. Fixed effects included intervention group (OWG vs. WR); timepoint (a 3-category variable indicating 0-, 3-, or 5.5-month timepoints); an interaction term between intervention group and timepoint to estimate intervention effects; and site, participant type, and cohort as data were clustered within these variables. Individual participants were considered as random effects in the model. Statistical significance was assessed using α = 0.05; all tests were two-sided. A mixed models analysis was performed using R package “GLMMadaptive” (R software version 4.0.0).

The zero inflation part of the model was formulated via logistic regression on timepoint. This was included to account for a high prevalence of zero values for minutes of outdoor walking (30%, 22%, and 36% of values were 0 min at 0, 3, and 5.5 months, respectively). We reported incidence rate ratios (IRRs), reflecting incidence of outdoor walking minutes, and associated 95% confidence intervals (CIs). Model selection was based on fit statistics, including the likelihood ratio statistic, Akaike Information Criterion, and Bayesian Information Criterion. In alignment with an intention-to-treat primary analysis [33], we imputed missing observations for time spent walking outdoors and the number of valid wear days. This was based on the fully conditional specification model for missing data, where each incomplete variable is imputed by a separate model [68]. Twenty imputations were used [68, 69]. Independent variables were age, sex, 6MWT distance, MiniBESTest total score, and calendar month (1–12) in which the OWG began.

To address secondary objectives, we evaluated potential effect modification by initial frailty level (frail or pre-frail vs. not frail) and sex (male vs. female), and the influence of intervention dose (two variables indicating the number of outdoor walk group vs. weekly reminder sessions received) on outdoor walking activity.

For the secondary outcomes of walking capacity, health-promoting behavior, and successful aging, we adopted generalized linear models with general estimating equations (GEE) based on a normal distribution and an unstructured correlation matrix to account for clustering of observations [70]. Scores on the secondary outcome measures were transformed into a z-score at each timepoint to standardize scores based on the same value of the baseline mean, and baseline standard deviation (SD) pooled across intervention groups. For example, a z-score for a measure at 3 months was computed as: z-score_3mon_ = individual score_3mon_ – mean of scores_0mon_ / SD of scores_0mon_ pooled across intervention groups. We performed the analysis using proc genmod in SAS version 9.4.

For each secondary outcome, we built two GEE models to examine the effect of intervention group on change from 0 to 3 months, and 0 to 5.5 months. For each GEE model, we entered each relevant z-scored difference as the dependent variable. Independent variables included a clustered “measure” variable; intervention group (OWG vs. WR); an interaction term between intervention group and the measure variable; and site, participant type, and cohort as data were clustered within these variables. Once it was determined that the association of intervention with the difference outcomes did not vary by measure, the interaction term was removed from the model. For each secondary outcome, we reported the regression parameter for the association of intervention group and the z-scored difference outcome (0–3 month and 0-5.5 month change) along with the associated 95% CI. Summary models were run with listwise deletion and interpretation was verified with results from models employing last value carried forward or last value carried backward to impute for missing data and after removing extreme scores to improve normalization of residuals. Because interpretation was similar, we reported results from the analysis with no imputation. We did not analyze scores on select measures (i.e., CHAMPS-outdoors, patient generated index, blood pressure, heart rate) specified in the protocol [31] as they conceptually duplicated other measures modeled.

Due to insufficient space, the 6MWT was administered on a shorter walkway than specified in the protocol for some individuals. Given walkway length affects 6MWT performance [71], we verified the results of modeling the outcome of walking capacity by conducting the analysis with and without individuals whose walkway distance changed from 0 to 3 months (*n* = 19) and 0–5 months (*n* = 17). Because interpretation was similar, we reported results based on the complete dataset.

## Results

### Characteristics of study sample

**Fig. 1:**
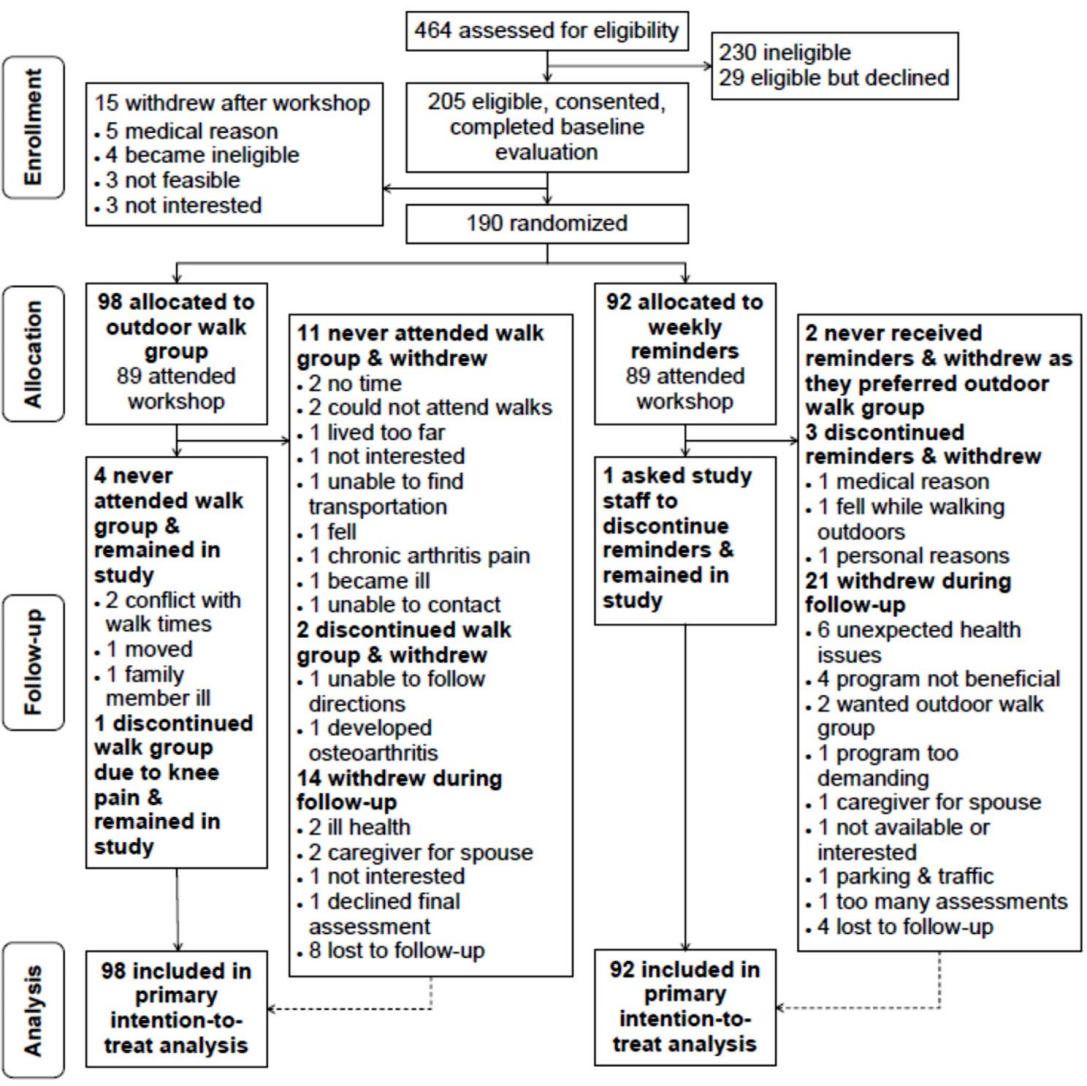
CONSORT diagram

Figure 1 presents the CONSORT diagram. Between February of 2018 and May of 2019, we enrolled 205 people who completed a baseline evaluation of which 15 withdrew prior to randomization mainly due to a medical reason or change in eligibility. After the workshop, we randomized 190 individuals to receive either the OWG (*n* = 98) or the WR (*n* = 92). Of the 190 participants, 34% and 66% were enrolled in cohort 1 (2018-19) and cohort 2 (2019-20), respectively. Thirty-six people (19%) participated with a partner. The top three reasons why participants experienced difficulty walking outdoors were health issues (e.g., arthritis/pain, fatigue, balance; 61%); inclement weather (41%); and low motivation (35%).

Table 1 presents baseline sociodemographic and health-related characteristics of participants by intervention group and site. Participants in each group were similar. At baseline, the mean ± standard deviation age of participants in the OWG and WR group was 75.3 ± 6.9 years and 74.7 ± 7.3 years, respectively. Percentage with female sex in the OWG and WR group was 75.5% and 70.7%, respectively. Percentage using a walking aid daily was 28.6% in the OWG vs. 21.7% in the WR group. The percentage of participants that were pre-frail and frail was 59.0% and 9.5% in the OWG, respectively, and 57.8% and 4.6% in the WR group, respectively. The four most prevalent health conditions were: arthritis (67%); hypertension (45%); cataracts (30%); and impaired hearing (27%). Additional file 1 presents participant health conditions at baseline by intervention group and site.

**Table 1:** Sociodemographic and health characteristics of participants at baseline by intervention group and site (*n* = 190)^*^

Characteristic	Outdoor Walk Group Intervention						Weekly Reminders Intervention					
	*n*	Site 1 (*n* = 26)	Site 2 (*n* = 28)	Site 3 (*n* = 26)	Site 4 (*n* = 18)	Pooled (*n* = 98)	*n*	Site 1 (*n* = 25)	Site 2 (*n* = 25)	Site 3 (*n* = 24)	Site 4 (*n* = 18)	Pooled (*n* = 92)
Participant type	98						92					
Individual		20 (76.9)	24 (85.7)	22 (84.6)	12 (66.7)	78 (79.6)		19 (76.0)	23 (92.0)	22 (91.6)	12 (66.7)	76 (82.6)
Dyad		6 (23.1)	4 (14.3)	4 (15.4)	6 (33.3)	20 (20.4)		6 (24.0)	2 (7.7)	2 (8.3)	6 (33.3)	16 (17.2)
Dyad type	20						16					
Spouse		6 (100)	4 (100)	2 (50.0)	2 (33.3)	14 (70.0)		6 (100.0)	0 (0)	2 (100)	4 (66.7)	12 (75.0)
Friend		0 (0)	0 (0)	2 (50.0)	4 (66.7)	6 (30.0)		0 (0)	2 (100)	0 (0)	2 (33.3)	4 (25.0)
Cohort, n	98						92					
2018-19		11 (42.3)	7 (25.0)	9 (34.6)	6 (33.3)	33 (33.7)		9 (36.0)	8 (32.0)	11 (45.8)	4 (22.2)	32 (34.8)
2019-20		15 (57.7)	21 (75.0)	17 (65.4)	12 (66.7)	65 (66.3)		16 (64.0)	17 (68.0)	13 (54.2)	14 (77.8)	60 (65.2)
Age in years, Mean (SD) Range	98	74.7 (6.9) 65–94	73.5 (6.3) 65–86	77.6 (7.1) 65–91	76.7 (6.6) 67–90	75.3 (6.9) 65–94	92	70.9 (4.0) 64–79	73.7 (6.4) 66–86	80.3 (8.3) 66–93	73.7 (7.1) 63–84	74.2 (7.4) 63–93
Sex												
Female	98	18 (69.2)	20 (71.4)	23 (88.5)	13 (72.2)	74 (75.5)	92	17 (68.0)	17 (68.0)	18 (75.0)	13 (72.2)	65 (70.7)
Male		8 (30.8)	8 (28.6)	3 (11.5)	5 (27.8)	24 (24.5)		8 (32.0)	8 (32.0)	6 (25.0)	5 (27.8)	27 (29.3)
BMI in kg/m^2^, Mean (SD) Range	98	29.6 (6.4) 18.6–49.3	31.8 (6.8) 18.8–49.3	26.8 (5.1) 19.3–36.4	29.3 (6.3) 20.1–40.8	29.6 (6.4) 18.6–49.3	90	28.6 (3.9) 18.6–35.4	29.9 (7.4) 15.6–44.7	27.8 (5.3) 20.4–45.3	32.1 (7.7) 21.8–49.4	29.3 (6.2) 15.6–49.4
Highest education level	98						92					
Secondary or lower		3 (11.5)	7 (25.0)	7 (26.9)	4 (22.2)	21 (21.4)		5 (20.0)	6 (24.0)	4 (16.7)	3 (16.7)	18 (19.6)
College diploma or taken college or university courses		10 (38.5)	10 (35.7)	9 (34.6)	5 (27.8)	34 (34.7)		5 (20.0)	8 (32.0)	11 (45.8)	10 (55.6)	34 (37.0)
University degree (undergraduate or graduate)		13 (50.0)	11 (39.3)	10 (38.5)	9 (50.0)	43 (43.9)		15 (60.0)	11 (44.0)	9 (37.5)	5 (27.8)	40 (43.5)
Marital status currently	98						91					
Single		3 (11.5)	3 (10.7)	6 (23.1)	2 (11.1)	14 (14.3)		4 (16.0)	2 (8.3)	5 (20.8)	1 (5.6)	12 (13.2)
Married		15 (57.7)	13 (46.4)	5 (19.2)	8 (44.4)	41 (41.8)		15 (60.0)	12 (50.0)	10 (41.7)	11 (61.1)	48 (52.7)
Widowed		4 (15.4)	9 (32.1)	9 (34.6)	3 (16.7)	25 (25.5)		3 (12.0)	8 (33.3)	5 (20.8)	5 (27.8)	21 (23.1)
Divorced/separated		4 (15.4)	3 (10.7)	6 (23.1)	5 (27.8)	18 (18.4)		3 (12.0)	2 (8.3)	4 (16.7)	1 (5.6)	10 (11.0)
Employed	97						92					
Yes		3 (11.5)	1 (3.6)	4 (15.4)	1 (5.9)	9 (9.3)		1 (4.0)	3 (12.0)	3 (12.5)	0 (0)	7 (7.6)
No		1 (3.8)	1 (3.6)	0 (0)	0 (0)	2 (2.1)		0 (0)	0 (0)	0 (0)	0 (0)	0 (0)
Retired		22 (84.6)	26 (92.9)	22 (84.6)	16 (94.1)	86 (88.7)		24 (96.0)	22 (88.0)	21 (87.5)	18 (100)	85 (92.4)
End of month finances	91						87					
Some money left over		20 (87.0)	23 (92.0)	19 (73.1)	15 (88.2)	77 (84.6)		18 (78.3)	20 (83.3)	18 (75.0)	14 (87.5)	70 (80.5)
Just enough to make ends meet		3 (13.0)	2 (8.0)	6 (23.1)	2 (11.8)	13 (14.3)		4 (17.4)	3 (12.5)	6 (25.0)	2 (12.5)	15 (17.2)
Not enough to make ends meet		0 (0)	0 (0)	1 (3.8)	0 (0)	1 (1.1)		1 (4.3)	1 (4.2)	0 (0)	0 (0)	2 (2.3)
Owns a car	98	19 (73.1)	27 (96.4)	18 (69.2)	17 (94.4)	81 (82.7)	92	24 (96.0)	25 (100)	18 (75.0)	18 (100)	85 (92.4)
Usual transportation to places too far to walk	98						91					
Takes own car		19 (73.1)	27 (96.4)	16 (61.5)	15 (83.3)	77 (78.6)		22 (88.0)	20 (83.3)	16 (66.7)	17 (94.4)	75 (82.4)
Family member or friend drives		2 (7.7)	1 (3.6)	2 (7.7)	3 (16.7)	8 (8.2)		0 (0)	3 (12.5)	5 (20.8)	1 (5.6)	9 (9.9)
Public transit		5 (19.2)	0 (0)	2 (7.7)	0 (0)	7 (7.1)		3 (12.0)	0 (0)	2 (8.3)	0 (0)	5 (5.5)
Adapted transportation		0 (0)	0 (0)	6 (23.1)	0 (0)	6 (6.1)		0 (0)	1 (4.2)	1 (4.2)	0 (0)	2 (2.2)
NEWS, Median (P_25_, P_75_)	98											
Residential density		221.0 (177.0, 320.0)	221.5 (177.0, 324.5)	423.5 (250.0, 475.0)	208.0 (177.0, 313.0)	252.5 (177.0, 443.0)		210.0 (187.0, 235.0)	213.0 (187.0, 338.0)	390.0 (201.5, 498.5)	198.0 (187.0, 258.0)	221.0 (187.0, 342.5)
Land-use mix-diversity		2.5 (2.1, 3.0)	2.2 (1.8, 3.1)	2.7 (1.9, 3.4)	2.1 (1.5, 3.1)	2.4 (1.9, 3.1)		2.7 (2.1, 3.4)	2.4 (1.7, 3.1)	2.6 (1.9, 3.1)	2.1 (1.7, 3.0)	2.4 (1.9, 3.1)
Land-use mix-access		2.8 (2.3, 3.5)	3.0 (2.3, 3.3)	3.1 (2.8, 4.0)	2.5 (1.8, 2.8)	2.8 (2.3, 3.4)		3.3 (2.3, 3.8)	3.3 (2.3, 3.5)	2.8 (2.0, 3.4)	2.8 (1.8, 3.7)	3.0 (2.3, 3.8)
Street connectivity		2.7 (2.0, 3.3)	3.2 (3.0, 4.0)	3.2 (3.0, 3.7)	3.0 (2.7, 3.0)	3.0 (2.5, 3.7)		3.0 (2.7, 3.3)	3.0 (2.7, 3.7)	2.5 (2.0, 3.0)	2.8 (2.3, 3.0)	2.8 (2.3, 3.3)
Infrastructure and safety for walking		3.2 (2.8, 3.5)	3.0 (2.3, 3.4)	3.1 (2.8, 3.4)	2.8 (2.1, 3.1)	3.0 (2.5, 3.4)		3.1 (2.8, 3.3)	3.0 (2.6, 3.4)	2.9 (2.7, 3.3)	2.6 (2.3, 3.1)	2.9 (2.6, 3.4)
Aesthetics		3.3 (2.8, 3.7)	3.2 (2.8, 3.7)	3.2 (2.8, 3.5)	3.0 (2.8, 3.5)	3.2 (2.8, 3.6)		3.2 (2.5, 3.5)	3.3 (2.7, 3.7)	3.0 (2.7, 3.3)	3.2 (2.8, 3.7)	3.2 (2.7, 3.7)
Traffic hazards		2.0 (1.5, 2.3)	2.3 (1.7, 2.7)	2.3 (2.0, 2.8)	2.0 (1.7, 2.5)	2.2 (1.7, 2.7)		2.2 (1.7, 2.3)	2.3 (1.7, 2.8)	2.0 (1.8, 2.4)	1.7 (1.2, 2.2)	2.2 (1.5, 2.5)
Crime		1.8 (1.3, 2.0)	1.8 (1.3, 2.0)	1.9 (1.0, 2.3)	1.3 (1.0, 1.5)	1.6 (1.3, 2.0)		1.8 (1.5, 2.3)	1.8 (1.3, 2.0)	1.4 (1.0, 2.1)	1.3 (1.0, 1.5)	1.5 (1.3, 2.3)
Lack of parking		2.0 (1.0, 3.0)	1.0 (1.0, 2.3)	3.0 (2.0, 3.0)	1.0 (1.0, 2.0)	2.0 (1.0, 3.0)		2.0 (1.0, 3.0)	2.0 (1.0, 3.0)	2.0 (1.0, 3.0)	1.0 (1.0, 1.0)	2.0 (1.0, 3.0)
Lack of cul-de-sacs		3.0 (2.0, 4.0)	3.0 (2.0, 4.0)	4.0 (3.0, 4.0)	3.0 (1.0, 3.0)	3.0 (2.0, 4.0)		2.0 (2.0, 4.0)	3.0 (2.0, 4.0)	3.0 (3.0, 4.0)	3.0 (2.0, 4.0)	3.0 (2.0, 4.0)
Hilliness		1.0 (1.0, 2.0)	1.0 (1.0, 1.0)	1.5 (1.0, 2.0)	1.0 (1.0, 2.0)	1.0 (1.0, 2.0)		1.0 (1.0, 2.0)	1.0 (1.0, 2.0)	2.0 (1.0, 2.3)	1.0 (1.0, 2.0)	1.0 (1.0, 2.0)
Physical barriers		1.0 (1.0, 2.0)	1.0 (1.0, 1.0)	1.0 (1.0, 2.0)	1.0 (1.0, 2.0)	1.0 (1.0, 2.0)		1.0 (1.0, 2.0)	1.0 (1.0, 2.0)	1.0 (1.0, 2.0)	1.0 (1.0, 1.0)	1.0 (1.0, 2.0)
Social interaction while walking		3.0 (3.0, 4.0)	3.0 (3.0, 4.0)	3.0 (2.3, 4.0)	3.0 (2.0, 3.0)	3.0 (3.0, 4.0)		3.0 (2.0, 3.0)	3.0 (2.0, 4.0)	3.0 (2.8, 3.0)	3.0 (2.0, 3.0)	3.0 (2.0, 3.0)
Smoker	98						92					
Current smoker		0 (0)	1 (3.6)	0 (0)	1 (5.6)	2 (2.0)		0 (0)	4 (16.0)	0 (0)	1 (5.6)	5 (5.4)
Ex-smoker		13 (50.0)	13 (46.4)	9 (34.6)	10 (55.6)	45 (45.9)		9 (36.0)	12 (48.0)	13 (54.2)	9 (50.0)	43 (46.7)
Never smoked		13 (50.0)	14 (50.0)	17 (65.4)	7 (38.9)	51 (52.0)		16 (64.0)	9 (36.0)	11 (45.8)	8 (44.4)	44 (47.8)
Wears glasses when walking	95	19 (76.0)	19 (67.9)	12 (50.0)	14 (77.8)	64 (67.4)	86	15 (65.2)	12 (48.0)	11 (52.4)	10 (58.8)	48 (55.8)
Charlson Comorbidity Index, Mean (SD) Range	98	2.05 (2.01) 0–11	1.93 (1.46) 0–5	2.35 (2.58) 0–11	1.71 (1.36) 0–5	2.05 (2.01) 0–11	92	2.12 (1.83) 0–7	1.84 (1.37) 0–4	2.33 (2.43) 0–9	1.28 (1.13) 0–4	1.93 (1.80) 0–9
Walking aid used daily	98						92					
None		20 (76.9)	22 (78.6)	13 (50.0)	15 (83.3)	70 (71.4)		22 (88.0)	21 (84.0)	14 (58.3)	15 (83.3)	72 (78.3)
Single point cane		4 (15.4)	4 (14.3)	6 (23.1)	2 (11.1)	16 (16.3)		2 (8.0)	2 (8.0)	5 (20.8)	2 (11.1)	11 (12.0)
Walking poles		1 (3.8)	1 (3.6)	1 (3.8)	1 (5.6)	4 (4.1)		1 (4.0)	1 (4.0)	1 (4.2)	0 (0)	3 (3.3)
4-wheeled walker		1 (3.8)	1 (3.6)	6 (23.1)	0 (0)	8 (8.2)		0 (0)	1 (4.0)	4 (16.7)	1 (5.6)	6 (6.5)
Frailty classification	95						87					
Not frail		10 (38.5)	13 (46.4)	4 (16.0)	3 (18.8)	30 (31.6)		13 (52.0)	10 (43.5)	6 (26.1)	4 (25.0)	33 (38.0)
Pre-frail		16 (61.5)	11 (39.3)	17 (68.0)	12 (75.0)	56 (59.0)		12 (48.0)	12 (52.2)	14 (60.9)	12 (75.0)	50 (57.8)
Frail		0 (0)	4 (14.3)	4 (16.0)	1 (6.3)	9 (9.5)		0 (0)	1 (4.4)	3 (13.0)	0 (0)	4 (4.6)

^*^All values are n (%) unless otherwise stated

### Intervention dose and fidelity

Among participants randomized to the OWG, 91% attended the workshop. Of the 98 participants in the OWG, 15% attended 0 OWG sessions (see Fig. 1 for reasons); 17% attended 1–8 sessions; and 67% attended 9–20 sessions. Reasons participants provided for absences included vacation/travel (31.7%), other commitments (e.g., personal reasons, friends/family, work/volunteering; 25.3%), medical (e.g., appointments, procedures/surgery; 19.6%), illness (10.0%), musculoskeletal issue (e.g., sore hip/knee; 6.1%), transportation (e.g., car issue, no transportation; 5.7%), OWG schedule (1.4%), and cold weather (0.4%). Of the 12 OWGs run during the intervention period across study sites, 83% of groups delivered 17–20 sessions.

Outdoor walk groups were run as planned. The mean percentage of sessions implementing each walk group component (e.g., warmup, continuous distance walks, skill-building activity) ranged from 95 to 99%. Of the 24 OWG sessions that were cancelled across sites, the reason for cancellation was rain (79%), poor air quality (13%), and high wind (8%). Forty-three OWG participants from all sites who attended sessions in week 3 and week 9 provided accelerometry and GPS data during the two continuous distance walks. Median values at week 3 vs. week 9 were as follows: walk 1 gait speed, 0.65 vs. 0.75 m/s; walk 2 gait speed, 0.61 vs. 0.77 m/s; walk 1 distance, 403 vs. 478 m; walk 2 distance, 391 vs. 431 m. Walk groups ran June to August except for the one site that ran three groups August to early October.

Among participants randomized to the WR group, 97% attended the workshop. Of the 92 participants in the WR group, 85% received 9 or 10, 13% received 2–8 reminders, and 2% received 0 out of 10 reminders.

### Primary outcome

Table 2 summarizes minutes spent in outdoor walking activity derived from accelerometry and GPS data by intervention group and timepoint, and the number of valid days the devices were worn. Although the median time spent walking outdoors was similar in each group at baseline (22.56 vs. 24.00 min in OWG and WR group, respectively), it decreased to 13.04 min in the OWG and increased to 26.07 min in the WR group at 3 months. At 5.5 months, median outdoor walking time in the OWG and WR group was again similar (median = 0 min in each group).

**Table 2:** Outdoor walking activity and accelerometer and GPS wear-time by intervention group and timepoint (non-imputed data)

Variable and Timepoint	Outdoor Walk Group Intervention (*n* = 98)		Weekly Reminders Intervention (*n* = 92)	
	*n*	Median (*P*_25_, *P*_75_)	*n*	Median (*P*_25_, *P*_75_)
Outdoor walking minutes				
0 months	86	22.56 (0, 67.04)	86	24.00 (0, 50.08)
3 months	60	13.04 (0, 43.47)	58	26.07 (0, 66.38)
5.5 months	61	0 (0, 23.00)	50	0 (0, 21.00)
No. valid days devices worn				
0 months	86	8.0 (7.0, 8.0)	86	8.0 (6.0, 8.0)
3 months	60	8.0 (7.0, 8.0)	58	8.0 (7.0, 8.0)
5.5 months	61	8.0 (7.0, 8.0)	50	8.0 (7.0, 8.0)

Abbreviations: GPS, global positioning system; P_25_, 25th percentile; P_75_, 75th percentile; min, minutes; No., number

Table 3 shows results from the zero-inflated, negative binomial regression modelling. After adjusting for site, participant type, and cohort, the change from baseline in the number of minutes spent walking outdoors was lower among people in the OWG compared to the WR program based on the IRR but this was not statistically significant. The IRR (95% CI) for the between-group change in outdoor walking activity from baseline to immediately post-intervention, and baseline to 5.5 months, was 0.74 (0.47, 1.14) and 0.77 (0.44, 1.34), respectively.

**Table 3:** Estimated rate ratios from negative binomial regression model of outdoor walking activity using imputed dataset

Independent Variable	Incidence rate ratio (95% CI)
**Site**	
1	Reference
2	0.69 (0.48, 0.98)
3	0.67 (0.46, 0.97)
4	0.69 (0.46, 1.04)
**Participant Type**	
Individual	Reference
Dyad	0.76 (0.53, 1.10)
**Cohort**	
1 (2018-19)	Reference
2 (2019-20)	1.16 (0.88, 1.52)
**Time**	
Baseline	Reference
Post-intervention	1.23 (0.91, 1.66)
Follow-up	0.83 (0.55, 1.26)
**Group**	
Weekly reminders	Reference
Outdoor walk group	1.17 (0.84, 1.62)
Group*Time	
0 months	Reference
3 months	0.74 (0.47, 1.14)
5.5 months	0.77 (0.44, 1.34)

Abbreviations: CI, confidence interval

Results from the secondary analysis showed that individuals who were frail or pre-frail spent significantly less time walking outdoors than those who were not frail (IRR = 0.68, 95% CI 0.52, 0.89). Thus, we tested for effect modification but the interaction term consisting of frailty level and intervention group was not significant (IRR = 0.72, 95% CI 0.43, 1.22). Neither sex (IRR = 1.25, 95% CI 0.92, 1.68) nor protocol adherence (OWG sessions received: IRR = 1.00, 95% CI 0.96, 1.03; WR received: IRR = 1.00, 95% CI 0.90, 1.12) was associated with time spent walking outdoors.

### Secondary outcomes

Table 4 summarizes performance on measures of walking capacity, health-promoting behavior, and successful aging by intervention group and timepoint; and results of the GEE modelling. After accounting for site, participant type, and cohort, there was significantly greater improvement in walking capacity from 0 to 3 months in the OWG compared to the WR group (beta for z-scored difference outcome (95% CI) = 0.14 (0.02, 0.26)). All other comparisons were not statistically significant. To understand the drivers of improved walking capacity, we re-ran the models for walking capacity for each underlying measure using proc GLM. The beta for the z-scored difference, representing the effect of the OWG compared to WR, for each measure of walking capacity, were as follows: mini-BESTest (beta = 0.11, *p* = 0.30, 95% CI -0.10, 0.31); 30STS (beta = 0.17, *p* = 0.10, 95% CI -0.03, 0.38); 10mWT (beta = 0.16, *p* = 0.22, 95% CI -0.09, 0.41); 6MWT (beta=-0.06, *p* = 0.61, 95% CI -0.30, 0.18); ASCQ (beta = 0.37, *p* = 0.00, 95% CI 0.12, 0.61).

**Table 4:** Performance and estimates of intervention effect on secondary outcome measures (non-imputed data)

Outcome *Component measures* (scoring)	Outdoor Walk Group Intervention (*n* = 98^*^) Mean (SD)			Weekly Reminders Intervention (*n* = 92^*^) Mean (SD)			Effect of Intervention on Change in Outcome β_z−scored difference_ (95% CI)^†^	
	0 months	3 months	5.5 months	0 months	3 months	5.5 months	0–3 month change	0-5.5 month change
Walking capacity							0.14 (0.02, 0.26)	0.03 (-0.12, 0.19)^‡^
*MiniBESTest* (0–28)	20.2 (5.0)	20.8 (5.4)	20.9 (5.7)	20.6 (5.5)	21.3 (4.6)	21.8 (5.2)		
*30-second sit-to-stand test* (# sit-to-stands)	8.1 (3.7)	9.3 (4.3)	9.4 (4.2)	8.6 (4.4)	9.6 (4.3)	10.1 (4.5)		
*10mWT* (m/s)	1.08 (0.24)	1.18 (0.21)	1.14 (0.24)	1.06 (0.22)	1.12 (0.24)	1.11 (0.26)		
*6MWT* (m)	359.0 (88.5)	393.9 (97.6)	373.2 (118.4)	357.9 (94.3)	399.8 (101.9)	364.5 (118.6)		
*ASCQ* (0–10)	7.8 (1.7)	8.2 (1.4)	8.2 (1.5)	8.0 (1.5)	7.9 (1.8)	7.8 (2.1)		
Health-promoting behavior							0.04 (-0.14, 0.23)^‡^	0.11 (-0.09, 0.32)^‡^
*Moderate-to-vigorous physical activity* (min/day)	58.5 (38.0)	48.8 (31.3)	41.4 (24.4)	58.1 (38.6)	59.8 (38.1)	48.0 (29.3)		
*LSA* (0-120)	63.3 (20.6)	70.1 (18.9)	64.8 (18.1)	65.6 (19.8)	70.7 (19.1)	62.9 (19.6)		
Successful aging							0.05 (-0.11, 0.22)	-0.07 (-0.25, 0.11)
*CHAMPS total score* (hours per week)	35.1 (17.2)	41.8 (19.1)	37.2 (17.0)	34.1 (15.9)	37.5 (17.5)	34.7 (15.0)		
*RAND-36 Emotional well-being* (0-100)	75.4 (16.9)	78.3 (15.1)	76.2 (17.0)	76.3 (14.0)	78.1 (16.0)	77.8 (15.3)		
*RAND-36 General health* (0-100)	64.1 (19.4)	62.7 (19.6)	63.4 (19.9)	64.3 (17.0)	64.5 (18.1)	64.0 (18.3)		

Abbreviations: SD, standard deviation; CI, confidence interval; 10mWT, 10-metre walk test (comfortable pace); m/s, meters/second; 6MWT, 6-minute walk test; m, meters; ASCQ, ambulatory self-confidence questionnaire; min, minutes; LSA, lifespace assessment; CHAMPS, community health activities model program for seniors; RAND, research and development

^*^n is the number of individuals, with varying numbers of measures within each individual

^†^Covariates in the model include site, participant type, and cohort

^‡^Association between intervention and secondary outcome z-score difference over time varied by component measure; thus, the summary beta is not representative of all measures

### Falls and adverse events

One serious adverse event occurred wherein a participant in the WR group experienced an injurious fall caused by a loss of balance while taking a walk outdoors. The participant suffered right arm abrasions and had some difficulty recalling the circumstances of the fall and was taken by ambulance to hospital.

There were five non-serious adverse events. Three events occurred during the intervention period. These included a non-injurious fall caused by a loss of balance when performing balance exercises during an OWG session wherein the person completed the OWG session; and two occurrences of hip pain as a result of over-exertion during OWG sessions reported by two people. The individuals with hip pain rejoined OWG sessions after skipping a session to rest. Outside of the intervention period, two falls occurred. One person in the OWG was returning home from a walk and slipped on an icy gravel walkway suffering knee abrasions; another person in the WR group slipped and fell but suffered no injury while walking on an icy walkway with a friend.

### Co-interventions

The percentage of individuals reporting participation in co-interventions (i.e., physical therapy, home and community exercise programs) in the OWG vs. WR group was 19% vs. 29%, respectively, at 3 months, and 35% vs. 32%, respectively, at 5 months. At the 3-month evaluation, the most common frequency of co-interventions was 2–3 times per week for 9% of people in the OWG, and 15% in the WR group. At 5.5-month evaluations, the most common frequency of co-interventions was 1 time per week for 17% of people in the OWG, and 2–3 times per week for 18% in the WR group.

## Discussion

A workshop and 10-week group, task-oriented OWG in parks was not superior to the workshop and 10 telephone WR in increasing outdoor walking activity in the short-term after accounting for site, participant type, and cohort, among older adults with difficulty walking outdoors. Sex, initial frailty level, and intervention dose received did not modify the influence of intervention group on time spent walking outdoors. The park-based OWG was superior to WR in improving walking capacity at 3 months (immediately post-intervention), but not at 5.5 months (2.5 months post-intervention), after accounting for site, participant type, and cohort. There was no difference between the OWG and WR group, however, in improving health-promoting behavior or successful aging in the short-term after accounting for site, participant type, and cohort. Given we targeted older adults with difficulty walking outdoors, it was not surprising that the percentage of study participants who were pre-frail (58.2%) was higher than in the general population (44.2% [12]). Almost all participants in the GO-OUT study had health conditions that were generally more prevalent than in the Canadian population [72] (e.g., GO-OUT vs. Canadian population aged 65 years and over: arthritis (67.4% vs. 46.5%), hypertension (45.3% vs. 43.8%), and cataracts (30.0% vs. 17.9%)). More than half of GO-OUT participants were pre-frail with leg strength, walking speed and walking endurance performance below normative values for healthy older adults [73].

Although a statistically significant effect of the OWG compared to WR on time spent walking outdoors was not observed, mean scores on measures of leg strength, comfortable walking speed, and walking endurance increased in both intervention groups, suggesting that physical capacity for walking improved. Balance, leg strength, walking self-efficacy, walking speed, and walking endurance are associated with not only outdoor walking activity [30], but also physical activity, participation, and perceived health status [74, 75] which underpinned secondary study outcomes of health-promoting behavior and successful aging. The small magnitude of improvements in balance, leg strength, walking self-efficacy, walking speed, and walking endurance in OWG members helps to explain why we did not observe an effect of the OWG on increasing outdoor walking, health-promoting behavior, and successful aging. Suboptimal attendance (did not attend or attended fewer than half of sessions) by a third of OWG participants, and, potentially, the higher proportion of frail or pre-frail individuals in the OWG versus WR group (68.4% vs. 62.0%), may explain why the OWG did not produce larger than observed changes in balance, leg strength, walking self-efficacy, walking speed, and walking endurance.

It is noteworthy that the OWG led to greater improvement in walking self-efficacy than WR in older adults with difficulty walking outdoors. In our qualitative process evaluation [76, 77], OWG participants described deriving increased walking confidence from multiple sources. First, participants in the OWG, but not the WR group, described improvements in walking distance, walking speed, ease of walking, leg strength, and fitness while walking in the park that increased confidence in their walking ability. Indeed, the walking distances and speeds achieved during OWG sessions increased throughout the 10-week program [78]. Participants frequently attributed their improved walking performance to the repetitive practice of varied outdoor walking tasks during supervised OWG sessions in the park that they would not normally attempt on their own. Second, some participants described how encouragement from the OWG leader to walk, for example, over challenging terrain (e.g., uneven ground) increased their confidence. Third, participants described a sense of well-being as their walking ability and health improved, and enjoyment during OWG sessions. They attributed the enjoyment to social interaction, facilitated by OWG leaders and assistants, in a group setting that increased their motivation to attend OWG sessions. Based on self-efficacy theory [79], the OWG program (but not WR) appears to have increased walking self-efficacy through three self-efficacy sources integral to the OWG program: performance accomplishments (successful performance of varied outdoor walking tasks); physiological signs and symptoms (walking practice in a safe, enjoyable environment); and verbal persuasion (encouragement of expert OWG facilitators).

Surprisingly, the unadjusted time spent walking outdoors decreased notably pre- to post-intervention in the OWG and was slightly improved in the WR group. Our qualitative process evaluation [76] showed that the scheduled and group nature of the OWG program promoted a sense of accountability to attend. Participants accomplished the goal of outdoor walking during the scheduled OWG program. Our qualitative findings [76] suggest this engagement did not translate to increased outdoor walking outside of the OWG program during the intervention period indicating that outdoor walking became less habitual. Thus, without the structure of the OWG, participants may have been less motivated to walk outdoors once the program finished. In contrast, WR participants may have maintained their outdoor walking activity pre- to post-intervention because of the consistent prompting to walk outdoors and review of physical activity guidelines and motivational strategies (e.g., SMART goals, pedometers). The Movingcall randomized trial [80] (288 adults, mean age: 42 years) showed that 12 coaching calls involving individualized goal setting, analysis of physical activity behaviour, and use of behaviour change techniques, were superior to a physical activity prescription in increasing moderate-to-vigorous physical activity. Limited-contact interventions deserve further attention in older adults as they provide a flexible option for people who prefer to exercise on their own and when physical distancing is required. Note that WR participants engaged in co-interventions more than OWG participants which may also help explain intervention effects at 3 months. At 5.5-month evaluations that occurred in October or November, at least half of participants in each group were not walking outdoors, likely because of colder weather, or snow or icy conditions [81].

### Implementation considerations

Although we did not observe benefits to outdoor walking behaviour, health-promoting behaviour or successful aging, other factors support the value of implementing the OWG in community settings. First, we showed that the OWG program can be implemented safely with a high level of fidelity in terms of completing each session component (i.e., warm-up, continuous distance walk, walking drills, continuous distance walk, cool down) for the targeted duration and walking distances, with appropriate adjustment to the ability level of participants [78]. Occurrence of serious adverse events in the GO-OUT study, specifically, an injurious fall that occurred while walking outdoors (0.5%), was low when compared to the rate of injurious falls of 7% estimated in community dwelling older adults in Canada [82]. The occurrence of non-serious adverse events during OWG sessions or outdoor walking, specifically non-injurious falls (1.6%) and hip pain (2.0%), is well below rates observed in older adults with COPD walking unsupervised on outdoor urban trails (falls: 10%; lower extremity pain: 32%) [23] and Finnish older adults completing a 12-month program of group, supervised, outdoor walking on a circular track that moved indoors during winter months (adverse events: mean 10.5%) [24].

Second, the OWG was beneficial in increasing walking self-efficacy which is expected to positively influence decisions to engage in walking and perceived health status in people with disability [75]. Third, the OWG program was successful with increasing the speed and distance of walking during sessions in a challenging park environment over time which suggests a benefit to those who attend. Suboptimal attendance among just under a third of OWG participants was primarily due to scheduling and health issues. Future research adapting OWG implementation for community settings should investigate whether knowing the OWG schedule in advance improves attendance. Fourth, older adults valued the social interaction facilitated by the group format, and described the OWG as enjoyable and fun [76]. Having OWG leaders and assistants with the ability to adapt to meet needs of pre-frail individuals as well as the OWG in a park environment was key to ensuring a safe, yet challenging, and enjoyable experience [76].

Exercise and physical activity programs targeting older adults achieving higher levels of attendance have shown health benefits. One trial [25] showed that group, supervised, Nordic pole walking on flat trails, and uphill and downhill in parks led to significantly greater gains in leg strength measured by the 30STS than indoor circuit training of balance, coordination and strength (both programs were three times a week for 12 weeks). Participants attended at least 70% of Nordic pole walking sessions. In the same study [25], indoor circuit training, where participants attended at least 80% of sessions, was superior to Nordic pole walking in improving balance measured by the one leg stance test.

### Strengths and limitations

Participants represented a distinct subgroup of the older adult population in terms of socioeconomic and health characteristics. The majority of participants owned a car and were female, retired, highly educated, and income sufficient. These participant characteristics may have resulted from the use of recruitment strategies (e.g., newspaper/website advertisements) that required a high degree of English literacy, and financial resources to pay for newspaper subscriptions or Internet access. Running the OWG at multiple parks may have dissuaded participation of individuals without access to a car. Thus, generalizability of findings to people with lower socioeconomic status is unclear. In future, trial design should incorporate available guidance to increase [83] and describe [84] trial diversity. The study was conducted in urban centres; thus, results may not apply to older adults living in rural areas due to differences in neighbourhood walkability that might indirectly influence outdoor walking [30]. There was a shortfall of participants. We could not recruit a third cohort due to COVID restrictions and a lack of resources. Recruiting additional participants might have changed the magnitude of the estimate of effect and improved the level of precision of the estimate of effect. At the design stage, we estimated sample size for a given power and hypothesized population effect size that was based on pilot study findings [28]. The effect size observed in the current study, however, was smaller than the hypothesized effect size. There is an inherent risk to using pilot data to estimate the population effect size for power calculations, but it was justified at the time due to the unavailability of estimates from similar populations in the literature. Strengths of the study included the high level of implementation fidelity in multiple urban centres, use of rigorous methods for accelerometry and GPS data collection and analysis [41], appropriate stratification that led to comparable groups at baseline, and accounting for data clustering at multiple levels (site, participant type, cohort) in the analysis.

## Conclusions

A group, task-oriented OWG in parks was superior to telephone WR in improving walking capacity through an increase in walking self-efficacy; however, it was not superior to WR in increasing outdoor walking activity in older adults with difficulty walking outdoors. Future research should focus on how to increase not only walking self-efficacy, but also balance, leg strength, and walking speed/endurance. Implementation of the GO-OUT program, specifically the workshop and OWG program, in community settings is warranted.

## Electronic supplementary material

Below is the link to the electronic supplementary material.

Supplementary Material 1

## Data Availability

The datasets generated and/or analysed during the current study are not publicly available as the participant consent forms did not address open public access to the data and due to limitations of the research ethics approval by the health sciences research ethics boards at the University of Toronto, University of Manitoba, University of Alberta and McGill University. Data are available upon request from the corresponding author on reasonable request and subject to research ethics boards review.

## Abbreviations

ASCQ: ambulatory self-confidence questionnaire
CCI: Charlson Comorbidity Index
COPD: chronic obstructive pulmonary disease
CHAMPS: community health activities model program for seniors
CI: confidence interval
GEE: general estimating equations
GO-OUT: getting older adults outdoors
GPS: global positioning system
HRQL: health-related quality of life
IRR: incidence rate ratio
LSA: life space assessment
LFE: low frequency extension
Mini-BESTest: Mini Balance Evaluation Systems test
NEWS: neighbourhood environment walkability scale
OWG: outdoor walk group
RAND: research and development
REDCap: research electronic data capture
6MWT: 6-minute walk test
SMART goals: specific measurable achievable realistic timely goals
SD: standard deviation
10mWT: 10-metre walk test
30STS: 30-second sit-to-stand test
WR: weekly reminders
