# Supplementary material for: Short-term effects of a park-based group mobility program on increasing outdoor walking in older adults with difficulty walking outdoors: the Getting Older Adults Outdoors (GO-OUT) randomized controlled trial
Source: BMC Geriatr. 2024 Sep 6;24:740. doi: 10.1186/s12877-024-05331-4 (PMC11378552; doi:10.1186/s12877-024-05331-4)
Supplement: Supplementary file 1 — Supplementary Material 1 [file 12877_2024_5331_MOESM1_ESM.docx]

**Additional file 1.**

**Supplemental Table 1: Participant health conditions by intervention group and site**

| **Health Conditions** | **Experimental** n (%) (n=98) | | | | | | **Control** n (%) (n=92) | | | | | |
| --- | --- | --- | --- | --- | --- | --- | --- | --- | --- | --- | --- | --- |
|  | **n** | **Site 1**  **(n=26)** | **Site 2**  **(n=28)** | **Site 3**  **(n=26)** | **Site 4**  **(n=18)** | **Pooled**  **(n=98)** | **n** | **Site 1**  **(n=25)** | **Site 2**  **(n=25)** | **Site 3**  **(n=24)** | **Site 4**  **(n=18)** | **Pooled**  **(n=92)** |
| No health conditions | 98 | 3 (11.5) | 1 (3.6) | 1 (3.8) | 1 (5.6) | 6 (6.1) | 92 | 2 (8.0) | 2 (8.0) | 1 (4.2) | 0 (0) | 5 (5.4) |
| Arthritis | 98 | 15 (57.7) | 23 (82.1) | 22 (84.6) | 10 (55.6) | 70 (71.4) | 92 | 13 (52.0) | 17 (68.0) | 17 (70.8) | 11 (61.1) | 58 (63.0) |
| Hypertension | 98 | 9 (34.6) | 15 (53.6) | 9 (34.6) | 8 (44.4) | 41 (41.8) | 92 | 12 (48.0) | 12 (48.0) | 12 (50.0) | 9 (50.0) | 45 (48.9) |
| Impaired hearing | 98 | 8 (30.8) | 7 (25.0) | 11 (42.3) | 6 (33.3) | 32 (32.7) | 92 | 2 (8.0) | 6 (24.0) | 9 (37.5) | 2 (11.1) | 19 (20.7) |
| Cataracts | 98 | 4 (15.4) | 11 (39.3) | 9 (34.6) | 5 (27.8) | 29 (29.6) | 92 | 4 (16.0) | 10 (40.0) | 9 (37.5) | 5 (27.8) | 28 (30.4) |
| Thyroid problem | 98 | 5 (19.2) | 7 (25.0) | 7 (26.9) | 6 (33.3) | 25 (25.5) | 92 | 6 (24.0) | 6 (24.0) | 6 (25.0) | 0 (0) | 18 (19.6) |
| Respiratory conditions (i.e., emphysema, COPD, bronchitis, asthma, pulmonary fibrosis) | 98 | 8 (30.8) | 5 (17.9) | 6 (23.1) | 5 (27.8) | 24 (24.5) | 92 | 3 (12.0) | 6 (24.0) | 2 (8.3) | 3 (16.7) | 14 (15.2) |
| Cancer | 98 | 5 (19.2) | 5 (17.9) | 9 (34.6) | 4 (22.2) | 23 (23.5) | 92 | 6 (24.0) | 2 (8.0) | 7 (29.2) | 0 (0) | 15 (16.3) |
| Diabetes | 98 | 4 (15.4) | 5 (17.9) | 5 (19.2) | 1 (5.6) | 15 (15.3) | 92 | 3 (12.0) | 7 (28.0) | 3 (12.5) | 1 (5.6) | 14 (15.2) |
| Heart attack | 98 | 3 (11.5) | 2 (7.1) | 3 (11.5) | 2 (11.1) | 10 (10.2) | 92 | 2 (8.0) | 0 (0) | 2 (8.3) | 3 (16.7) | 7 (7.6) |
| Stroke | 98 | 3 (11.5) | 1 (3.6) | 4 (15.4) | 2 (11.1) | 10 (10.2) | 92 | 0 (0) | 2 (8.0) | 2 (8.3) | 2 (11.1) | 6 (6.5) |
| Glaucoma | 98 | 1 (3.8) | 1 (3.6) | 3 (11.5) | 2 (11.1) | 7 (7.1) | 92 | 1 (4.0) | 5 (20.0) | 4 (16.7) | 1 (5.6) | 11 (12.0) |
| Orthopedic issue | 98 | 1 (3.8) | 2 (7.1) | 2 (7.7) | 1 (5.6) | 6 (6.1) | 92 | 0 (0) | 2 (8.0) | 0 (0) | 0 (0) | 2 (2.2) |
| Other cardiac conditions | 98 | 0 (0) | 2 (7.1) | 2 (7.7) | 1 (5.6) | 5 (5.1) | 92 | 1 (4.0) | 1 (4.0) | 2 (8.3) | 0 (0) | 4 (4.3) |
| Osteoporosis | 98 | 0 (0) | 2 (7.1) | 0 (0) | 2 (11.1) | 4 (4.1) | 92 | 0 (0) | 2 (8.0) | 0 (0) | 0 (0) | 2 (2.2) |
| Angina | 98 | 1 (3.8) | 0 (0) | 1 (3.8) | 1 (5.6) | 3 (3.1) | 92 | 1 (4.0) | 2 (8.0) | 0 (0) | 0 (0) | 3 (3.3) |
| Inflammatory bowel disease | 98 | 3 (11.5) | 0 (0) | 0 (0) | 0 (0) | 3 (3.1) | 92 | 0 (0) | 0 (0) | 2 (8.3) | 0 (0) | 2 (2.2) |
| Parkinson’s disease | 98 | 0 (0) | 0 (0) | 1 (3.8) | 2 (11.1) | 3 (3.1) | 92 | 2 (8.0) | 0 (0) | 0 (0) | 2 (11.1) | 4 (4.3) |
| Sleep apnea | 98 | 0 (0) | 2 (7.1) | 0 (0) | 1 (5.6) | 3 (3.1) | 92 | 0 (0) | 2 (8.0) | 0 (0) | 1 (5.6) | 3 (3.3) |
| Liver disease | 98 | 2 (7.7) | 0 (0) | 0 (0) | 0 (0) | 2 (2.0) | 92 | 0 (0) | 1 (4.0) | 0 (0) | 0 (0) | 1 (1.1) |
| Fibromyalgia | 98 | 0 (0) | 0 (0) | 0 (0) | 1 (5.6) | 1 (1.0) | 92 | 2 (8.0) | 0 (0) | 1 (4.2) | 0 (0) | 3 (3.3) |
| Hemiplegia | 98 | 1 (3.8) | 0 (0) | 0 (0) | 0 (0) | 1 (1.0) | 92 | 0 (0) | 1 (4.0) | 0 (0) | 0 (0) | 1 (1.1) |
| Other | 98 | 2 (7.7) | 6 (21.4) | 1 (3.8) | 6 (33.3) | 15 (15.3) | 92 | 2 (8.0) | 7 (28.0) | 2 (8.3) | 0 (0) | 11 (12.0) |

Abbreviations: COPD, chronic obstructive pulmonary disease.
